# Scoping review on the link between economic growth, decent work, and early childhood caries

**DOI:** 10.1186/s12903-023-03766-6

**Published:** 2024-01-13

**Authors:** Morẹ́nikẹ́ Oluwátóyìn Foláyan, Rosa Amalia, Arthur Kemoli, Imen Ayouni, Arthemon Nguweneza, Duangporn Duangthip, Ivy Guofang Sun, Jorma I. Virtanen, Ray M. Masumo, Ana Vukovic, Ola B. Al-Batayneh, Balgis Gaffar, Tshepiso Mfolo, Robert J. Schroth, Maha El Tantawi

**Affiliations:** 1 Early Childhood Caries Advocacy Group, Winnipeg, MB Canada; 2 Department of Child Dental Health, Obafemi Awolowo University, Ile-Ife, Nigeria; 3 Department of Preventive and Community Dentistry, Faculty of Dentistry, Universitas Gadjah Mada, Yogyakarta, Indonesia; 4 Department of Paediatric Dentistry and Orthodontics, University of Nairobi, Nairobi, Kenya; 5 Department of Pediatrics and Child Health, Faculty of Health Sciences, University of Cape Town, Cape Town, South Africa; 6 Division of Human Genetics, Department of Pathology, Faculty of Health Sciences, University of Cape Town, Cape Town, South Africa; 7 Faculty of Dentistry, The University of Hong Kong, Hong Kong, SAR China; 8 Department of Clinical Dentistry, University of Bergen, Bergen, Norway; 9 Department of Community Health and Nutrition, Tanzania Food and Nutrition Centre, Dar es Salaam, Tanzania; 10 Clinic for Pediatric and Preventive Dentistry, School of Dental Medicine, University of Belgrade, Belgrade, Serbia; 11 Department of Orthodontics, Pediatric and Community Dentistry, College of Dental Medicine, University of Sharjah, Sharjah, United Arab Emirates; 12 Department of Preventive Dentistry, Faculty of Dentistry, Jordan University of Science and Technology, Irbid, Jordan; 13 Department of Community Dentistry, University of Pretoria, Pretoria, South Africa; 14 Dr. Gerald Niznick College of Dentistry, University of Manitoba, Winnipeg, Canada; 15 Department of Pediatric Dentistry and Dental Public Health, Faculty of Dentistry, Alexandria University, Alexandria, Egypt

**Keywords:** Sustainable development, Dental caries, child, preschool, Economic development, Social justice, Employment, Right to work, Equality, Technological innovation, Entrepreneurship, Forced labor, Slavery, Human trafficking, Policy formulation

## Abstract

**Background:**

Early Childhood Caries (ECC) is a prevalent chronic non-communicable disease that affects millions of young children globally, with profound implications for their well-being and oral health. This paper explores the associations between ECC and the targets of the Sustainable Development Goal 8 (SDG 8).

**Methods:**

The scoping review followed the Preferred Reporting Items for Systematic Reviews and Meta-Analyses Extension for Scoping Reviews (PRISMA-ScR) guidelines. In July 2023, a search was conducted in PubMed, Web of Science, and Scopus using tailored search terms related to economic growth, decent work sustained economic growth, higher levels of productivity and technological innovation, entrepreneurship, job creation, and efforts to eradicate forced labor, slavery, and human trafficking and ECC all of which are the targets of the SDG8. Only English language publications, and publications that were analytical in design were included. Studies that solely examined ECC prevalence without reference to SDG8 goals were excluded.

**Results:**

The initial search yielded 761 articles. After removing duplicates and ineligible manuscripts, 84 were screened. However, none of the identified studies provided data on the association between decent work, economic growth-related factors, and ECC.

**Conclusions:**

This scoping review found no English publication on the associations between SDG8 and ECC despite the plausibility for this link. This data gap can hinder policymaking and resource allocation for oral health programs. Further research should explore the complex relationship between economic growth, decent work and ECC to provide additional evidence for better policy formulation and ECC control globally.

**Supplementary Information:**

The online version contains supplementary material available at 10.1186/s12903-023-03766-6.

## Introduction

Early Childhood Caries (ECC) is a dental condition that affects young children worldwide. Untreated ECC causes dental pain, infections, nutritional impairments, developmental delays, reduced quality of life, and increased healthcare costs for individuals and societies [1]. Defined as any carious lesion in the primary teeth of children under the age of 6 years, the impact of ECC on wellness and wellbeing is particularly significant among socially disadvantaged populations, thereby exacerbating oral health inequalities [2] With approximately 514 million affected children globally, ECC ranks among the most common childhood diseases [3, 4]. As global health priorities continue to evolve, addressing ECC within the context of the United Nations’ Sustainable Development Goal 8 (SDG8) becomes crucial, as this goal aims to promote sustained, inclusive, and sustainable economic growth, full and productive employment, and decent work for all. SDG8 emphasizes the importance of labor rights, eradicating modern slavery and child labor, and ensuring equal access to the benefits of entrepreneurship and innovation. In addition, it reiterates the value of the reciprocal links between social, environmental, and economic policies, full employment, and decent work.

Within the framework of SDG8, there is an opportunity to address the issue of untreated ECC using a human rights perspective [5, 6]. The high prevalence of ECC among socially disadvantaged children highlights the need to promote ECC management through the lens of social justice, health equity, and human rights [7, 8]. By linking macro-social development with meso- and micro-economic growth, we can potentially achieve a more equitable distribution of wealth and have a direct impact on health, including oral health [9]. SDG8 also encourages investments in health systems and infrastructure [10]. Incorporating oral health services into health systems and infrastructure can enhance preventive efforts and early intervention for ECC [11, 12]. This integration can lead to a more comprehensive approach to oral health care, aligning with the principles of SDG8 to ensure well-being for all.

SDG8 includes 12 targets, one of which is achieving full and productive employment, decent work for all, and equal pay for work of equal value (SDG8.5). Full and productive employment refers to the availability of quality job opportunities that enable individuals to earn a decent income and contribute to economic growth [5]. Decent work improves income stability and economic security, ultimately leading to greater household income and reduced income inequality [13]. Achieving equal pay for work of equal value is crucial for addressing gender discrimination in the labor market, which is particularly relevant for ECC since maternal socioeconomic status strongly influences the risk of ECC [14, 15]. Accomplishing SDG8.5 can enable households to meet their basic needs, access better healthcare and education, and invest in their future [16]. It will also lead to improved living standards, reduced poverty rates, enhanced economic resilience, and the creation of a more inclusive society [17, 18]. By using a rights-based approach, SDG8 aligns with the goal of achieving equitable access to health, including oral health, for all individuals.

Given that ECC is preventable adequate and timely preventive and prophylactic cost-effective programs, and in some cases, early lesions can be reversed with early detection and available treatment options, it is essential to include the management of untreated ECC on the global disease elimination agenda [6]. Treating dental caries, particularly in young children, can be expensive and time-consuming, leading families to miss work to address their child’s oral health needs, consequently affecting their economic productivity [19]. ECC is more prevalent in disadvantaged and vulnerable populations who frequently consume sugar, have poor access to adequate dental care and poor education on oral hygiene practices [20, 21]. This oral health disparity can contribute to broader health and well-being inequalities that the goals of SDG8 try to address. Conversely, poor economic development and growth can negatively affect the prevalence and severity of ECC. Poor economic growth and development reduces expenditure on health [22]. yet, higher expenditure on health may be associated with lower prevalence of ECC [23].

**Fig. 1:**
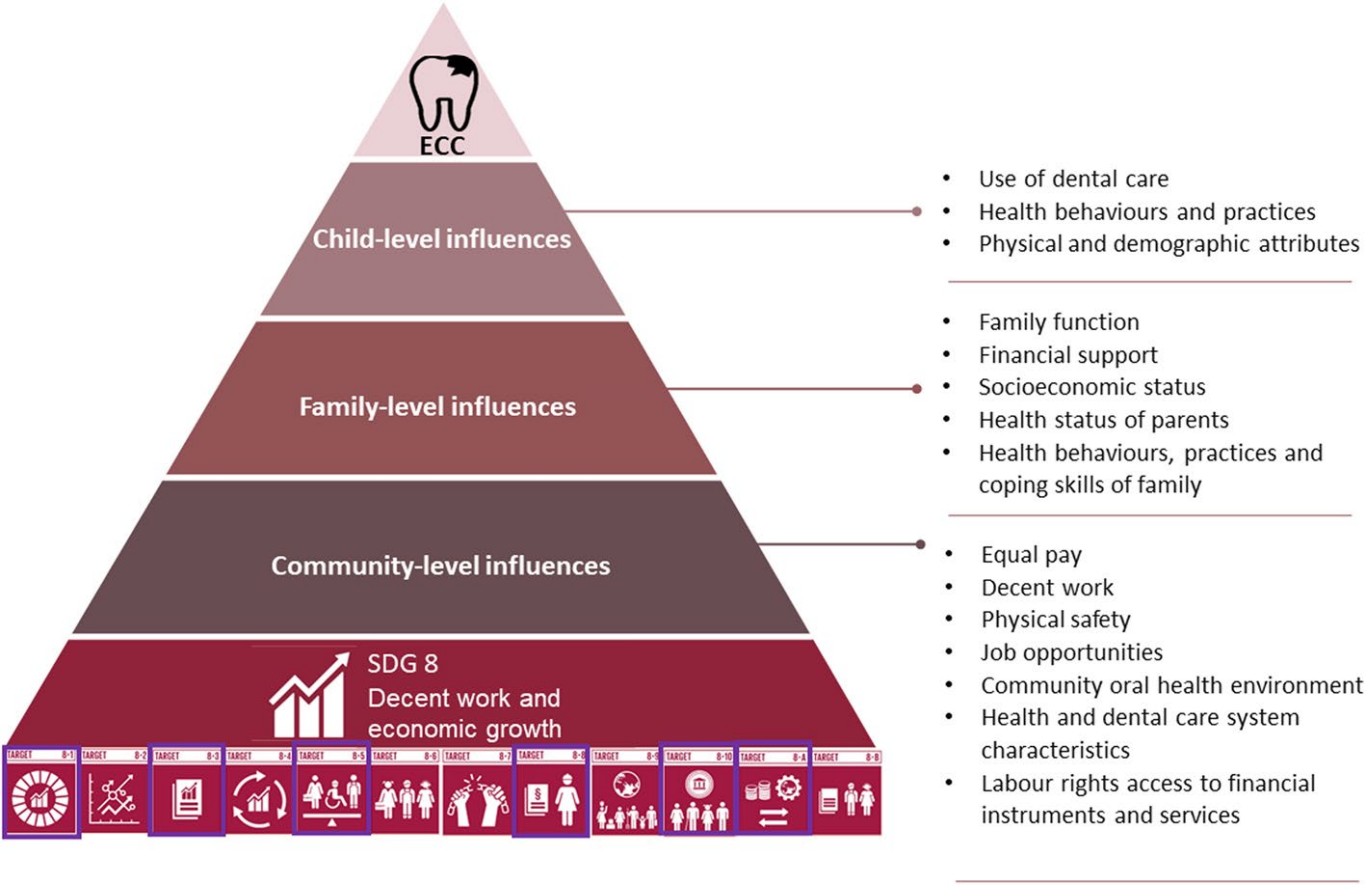
The conceptual framework of ECC and decent work and economic growth (SDG8) adaptation from Fisher-Owens Model [24]

By prioritizing the elimination of untreated ECC within the SDG8 framework, we can strive for a more equitable distribution of resources and higher household income. We conceptualized the impact of interventions related to SDG8 on ECC using the Fisher-Owen et al.’s 2007 model [24] depicted in Fig. 1. We perceive that at least, five targets of SDG8 could have a direct or indirect community-level, family-level, and child-level influences on the risk of ECC: SDG8.1 (sustainable economic growth), SDG8.3 (promote policies to support job creation and growing enterprises), SDG8.5 (full employment and decent work with equal pay), SDG8.8 (protection of labor rights and promotion of safe working environments), and SDG8.A (universal access to banking, insurance and financial services). The outcomes of SDG8 can indirectly reduce the risk of and global prevalence of ECC. The exploration of the intersection between ECC and SDG8 can help identify opportunities to leverage economic growth and employment opportunities to strengthen oral health systems.

Though there is very little known about the links between SDG8 and ECC, ecological studies suggest that a growth in per-capita gross national income was significantly associated with higher prevalence of ECC in children aged 36 to 71 months [25]. This association was found to be the reverse for children with ECC in European member countries [26] and for children in Serbia though the findings in Serbia was not statistically significant [27]. The aim of this scoping review was to map the evidence on the links between ECC and targets of the SDG8, and to identify research gaps to be filled to provide evidence on the link between SDG8 and ECC.

## Methods

We conducted this scoping review to explore the connections between ECC and the objectives of SDG8, which encompass economic growth and decent work. To ensure methodological rigor and transparency, we followed the Preferred Reporting Items for Systematic Reviews and Meta-Analyses Extension for Scoping Reviews (PRISMA-ScR) guidelines [28] during the review process.

### Research questions

The following questions guided this review: What is the existing evidence on the association between decent work and economic growth (sustained economic growth, higher levels of productivity and technological innovation, entrepreneurship, job creation, and efforts to eradicate forced labor, slavery, and human trafficking) and ECC?

### Search strategy

In January 2023, a search was conducted on three electronic databases: PubMed, Web of Science, and Scopus. The search utilized a combination of key terms as shown in Additional file 1: Appendix 1. The search terms were tailored to meet the specific requirements of each database. The key terms used were for the Pubmed search were: (((((((((“Economic Development”[Mesh]) OR “Sustainable Growth”[Mesh]) OR “Right to Work”[Mesh]) OR “Unemployment”[Mesh]) OR “Small Business”[Mesh]) OR “Human Trafficking”[Mesh]) OR “Labor Unions”[Mesh]) OR “Working Poor”[Mesh]) OR “Resource Allocation”[Mesh]) OR “Banking, Personal”[Mesh]. That for Web of Science search were: (((((((((“Economic Development”[Mesh]) OR “Sustainable Growth”[Mesh]) OR “Right to Work”[Mesh]) OR “Unemployment”[Mesh]) OR “Small Business”[Mesh]) OR “Human Trafficking”[Mesh]) OR “Labor Unions”[Mesh]) OR “Working Poor”[Mesh]) OR “Resource Allocation”[Mesh]) OR “Banking, Personal”[Mesh] and ((((((“Dental Caries”[Mesh]) OR “Tooth Demineralization”[Mesh]) OR (caries[Text Word])) OR (dental decay[Text Word])) OR (dental cavities [Text Word])) OR (tooth cavities[Text Word])) OR (enamel demineralization[Text Word]). Screening of publications was conducted from the inception of the databases up to 2023. The search was completed in July 2023.

### Eligibility criteria and article selection

For inclusion in this review, only English language publications until July 2023 were considered. The selected studies included cross-sectional, case-control, and cohort designs, and they reported findings on the association between decent work, economic growth, related factors, and ECC among children aged six years and below. To maintain the focus of this review on the association between decent work, economic growth-related factors, and ECC, studies that solely examined the prevalence and severity of ECC with no reference to the goals of SDG 8 were excluded. Publications that were not primary studies such as ecological studies and letters to the editors were also excluded.

The literature obtained from the database searches was exported to Zotero version 6, a reference management software. Duplicate publications were identified and removed using the “duplicate items” function in Zotero. Title and abstract screening were carried out by two independent reviewers (IA, AN) who followed the eligibility criteria established for this review. No attempts were made to contact authors or institutions for additional sources of information.

## Results

**Fig. 2:**
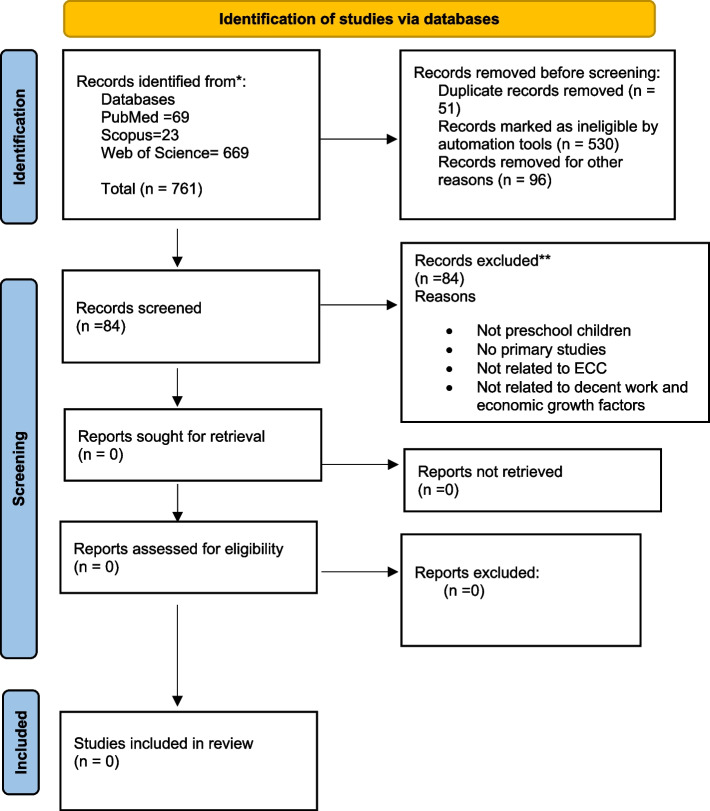
Flow diagram based on the Preferred Reporting Items for Systematic Reviews and Meta-Analyses 2020 flowchart template of the search and selected process

The initial search across three databases, namely PubMed, Web of Science, and Scopus, using the predefined search terms resulted in a total of 761 articles. After removing duplicates and ineligible manuscripts, 84 unique articles remained for further screening. However, none of the identified studies provided data on the association between decent work, economic growth-related factors, and ECC. Figure 2 shows the details of the search findings.

## Discussion

Recognizing the potential impact of socioeconomic development oral health is crucial, as it paves the way for a future where every child can access high-quality oral healthcare and enjoy a healthy and prosperous life. The SDG8 has the potential to contribute to global health and well-being. However, despite the plausible evidence supporting the link between SDG8 and ECC, this scoping review could identify no evidence derived from primary studies supporting this connection. The study finding suggest there is a lacuna of evidence derived from primary studies on the links between SDG8 and ECC.

This study represents the first comprehensive analysis examining the potential association between ECC and SDG8. It highlights the possibility of generating evidence to establish this link through further research. It is important to note that attributing the impact of economic development on ECC to SDG8 may be challenging due to links with other SDGs that can influence the prevalence, burden, and severity of ECC. Nevertheless, this challenge does not negate the potentials for developing new methodologies for assessing the impact of economic development on oral health in children. Perhaps as more countries undertake nationally representative oral health surveys and adopt SDG8 measurements, future investigations of potential interactions are possible.

There are numerous studies on the links between human health on health expenditure, economic activity and growth and the SDG8 [22, 29]. There are, however, fewer studies on the impact of oral health on economic activity and growth. One study suggests that poor oral health causes an indirect global loss worth $144 billion, direct annual cost of oral problems was about $298 billion [30]. There are no specific data on the impact of ECC and ECC expenditure on economic activity and growth despite the recognized economic toll ECC exerts [31]. The absence of specific data can significantly impact the ability of policymakers to establish relevant oral health programs, making it challenging to develop ECC-focused policies and effectively allocate resources for children’s oral health. Concrete data on the economic toll of ECC is crucial for designing sustainable oral health programs and promoting oral health in vulnerable populations.

There is a growing body of literature that explores the relationship between macroeconomic activities, economic growth, and population health [32, 33]. Economic growth has the potential to positively influence population health by promoting the utilization of preventive health services, improving nutrition, and reducing the risk of health disorders caused by diseases. However, empirical evidence on the impact of economic growth on population health is diverse and lacks a clear consensus [34]. This is reflected in the findings of the ecological studies on the impact of economic growth on the risk for ECC [23, 25–27] suggestive of differences in global and country-level findings on the impact of economic development on the risk of ECC.

In addition, a prior ecological study further puts a caveat to the possible impact of economic development on ECC wherein the gross national income per capita for females was associated with lower ECC prevalence [35]: countries with more females living under 50% of median income had higher prevalence of ECC among 3 to 5-year olds [36]; and the gross national income per capita for females had a great effect on ECC prevalence [35]. These studies underscore the need for further research and collaborative efforts among experts to gain a comprehensive understanding of the complex relationship between ECC and the SDG8 to promote population oral health in the context of economic growth. Without a concrete understanding of the relationship between economic growth and health, designing targeted and effective programs to address ECC becomes challenging.

Moreover, the absence of empirical evidence concerning the effective and efficient allocation of additional resources to promote oral health, specifically in preventing untreated ECC, creates a critical gap that requires attention. Without this evidence, there is a risk of misallocating resources and efforts, leading to inefficiencies in oral health programs. Consequently, preventive measures targeting ECC may not receive sufficient support, allowing the condition to persist and worsen [37]. The lack of data-driven insights may result in missed opportunities to implement innovative and effective strategies for ECC prevention. Promising interventions may not undergo adequate investigation, and their potential impact on preventing ECC might not be fully realized, especially when competing with other health priorities. Consequently, ECC prevention efforts may not receive the necessary attention and resources required to make a significant impact on children’s oral health [38].

Understanding this aspect will provide valuable insights for the development and implementation of oral health policies for children. Given the intricate relationship between SDG8 and health [39], as well as the close connection between oral health and overall health [40], it is reasonable to assume that SDG8 and oral health are intertwined. Therefore, empirical studies examining the link between economic development, decent workplaces, and the oral health of children are warranted.

The SDG 8 targets creates an opportunity to explore the possible impact of having a healthy workforce with decent work and economic growth. The provision of decent, healthy, and safe oral health workforce will help improve ECC outcomes. To quantify contributory benefits of decent work and economic growth on ECC indicators measuring this impact is needed as this evidence can encourage investments in enhancing working conditions and safeguarding oral health workers to tackle ECC.

In conclusion, although there are plausible links between SDG8 and ECC, there is currently no evidence derivable from primary studies showing these links. Though the evidence on the associations between SDG8 and health are controversial, these findings further substantiate the possibility to generate evidence on the associations between the SDG8 and ECC. Generating evidence on the links between SDG8 and oral health, inclusive of ECC, will help drive investments, policy formulation, and programs linking macrostructural factors to enhance the control of ECC globally.

### Supplementary Information

**Additional file 1: Appendix 1.**

## Data Availability

The datasets used and/or analysed for the study are publicly accessible.

## Abbreviations

ECC: Early Childhood Caries
PRISMA-ScR: Preferred Reporting Items for Systematic Reviews and Meta-Analyses Extension for Scoping Reviews guidelines
SDG: Sustainable Development Goal
