# Supplementary material for: Scoping review on the link between economic growth, decent work, and early childhood caries
Source: BMC Oral Health. 2024 Jan 13;24:77. doi: 10.1186/s12903-023-03766-6 (PMC10787988; doi:10.1186/s12903-023-03766-6)
Supplement: Supplementary file 1 — Additional file 1: Appendix 1. [file 12903_2023_3766_MOESM1_ESM.docx]

**Appendix 1**

**Scopus**

Bottom of Form

| History Count | Search Terms | Results |
| --- | --- | --- |
| 3 | ( TITLE-ABS-KEY ( ( {economic growth}  OR  {economic development policy}  OR  {employment policy}  OR  {inclusive economic growth}  OR  {sustainable growth}  OR  {economic development}  OR  {economic globalization}  OR  {economic globalisation}  OR  {economic productivity}  OR  {low-carbon economy}  OR  {inclusive growth}  OR  microfinanc*  OR  micro-financ*  OR  micro-credit*  OR  microcredit*  OR  {equal income}  OR  {equal wages}  OR  {decent job}  OR  {decent jobs}  OR  {quality job}  OR  {quality jobs}  OR  {job creation}  OR  {full employment}  OR  {employment protection}  OR  {informal employment}  OR  {precarious employment}  OR  {unemployment}  OR  {precarious job}  OR  {precarious jobs}  OR  microenterprise*  OR  micro-enterprise*  OR  {small enterprise}  OR  {medium enterprise}  OR  {small enterprises}  OR  {medium enterprises}  OR  {small entrepreneur}  OR  {starting entrepreneur}  OR  {medium entrepreneur}  OR  {small entrepreneurs}  OR  {medium entrepreneurs}  OR  {starting entrepreneurs}  OR  {social entrepreneurship}  OR  {safe working environment}  OR  {labor market institution}  OR  {labor market institutions}  OR  {labour market institution}  OR  {labour market institutions}  OR  {forced labour}  OR  {forced labor}  OR  {child labour}  OR  {child labor}  OR  {labour right}  OR  {labor right}  OR  {labour rights}  OR  {labor rights}  OR  {modern slavery}  OR  {human trafficking}  OR  {child soldier}  OR  {child soldiers}  OR  {global jobs}  OR  {living wage}  OR  {minimum wage}  OR  {circular economy}  OR  {inclusive economy}  OR  {rural economy}  OR  {Foreign Development Investment}  OR  {Aid for Trade}  OR  {trade unions}  OR  {trade union}  OR  {working poor}  OR  {Not in Education, Employment, or Training}  OR  {carbon offset}  OR  {carbon offsetting}  OR  {carbon offsets}  OR  {offset project}  OR  {offset projects}  OR  {economic diversification}  OR  {material footprint}  OR  {resource efficiency}  OR  ( {cradle to cradle}  AND  {economy} )  OR  {economic decoupling}  OR  {labour market disparities}  OR  {sustainable tourism}  OR  {ecotourism}  OR  {community-based tourism}  OR  {tourism employment}  OR  {sustainable tourism policy}  OR  {financial access}  OR  {financial inclusion}  OR  {access to banking} )  AND NOT  {health} ) )  AND  ( TITLE-ABS-KEY ( caries )  OR  TITLE-ABS-KEY ( dental  AND  caries )  OR  TITLE-ABS-KEY ( dental  AND  decay )  OR  TITLE-ABS-KEY ( dental  AND  cavities )  OR  TITLE-ABS-KEY ( enamel  AND  demineralization )  OR  TITLE-ABS-KEY ( tooth  AND  demineralization )  OR  TITLE-ABS-KEY ( tooth  AND  cavities ) ) View Less | [23](https://08105dxi5-1105-y-https-www-scopus-com.mplbci.ekb.eg/search/history/results.uri?origin=searchhistory&shid=3) |
| 2 | TITLE-ABS-KEY ( caries )  OR  TITLE-ABS-KEY ( dental  AND  caries )  OR  TITLE-ABS-KEY ( dental  AND  decay )  OR  TITLE-ABS-KEY ( dental  AND  cavities )  OR  TITLE-ABS-KEY ( enamel  AND  demineralization )  OR  TITLE-ABS-KEY ( tooth  AND  demineralization )  OR  TITLE-ABS-KEY ( tooth  AND  cavities ) | [125,261](https://08105dxi5-1105-y-https-www-scopus-com.mplbci.ekb.eg/search/history/results.uri?origin=searchhistory&shid=2) |
| 1 | TITLE-ABS-KEY ( ( {economic growth}  OR  {economic development policy}  OR  {employment policy}  OR  {inclusive economic growth}  OR  {sustainable growth}  OR  {economic development}  OR  {economic globalization}  OR  {economic globalisation}  OR  {economic productivity}  OR  {low-carbon economy}  OR  {inclusive growth}  OR  microfinanc*  OR  micro-financ*  OR  micro-credit*  OR  microcredit*  OR  {equal income}  OR  {equal wages}  OR  {decent job}  OR  {decent jobs}  OR  {quality job}  OR  {quality jobs}  OR  {job creation}  OR  {full employment}  OR  {employment protection}  OR  {informal employment}  OR  {precarious employment}  OR  {unemployment}  OR  {precarious job}  OR  {precarious jobs}  OR  microenterprise*  OR  micro-enterprise*  OR  {small enterprise}  OR  {medium enterprise}  OR  {small enterprises}  OR  {medium enterprises}  OR  {small entrepreneur}  OR  {starting entrepreneur}  OR  {medium entrepreneur}  OR  {small entrepreneurs}  OR  {medium entrepreneurs}  OR  {starting entrepreneurs}  OR  {social entrepreneurship}  OR  {safe working environment}  OR  {labor market institution}  OR  {labor market institutions}  OR  {labour market institution}  OR  {labour market institutions}  OR  {forced labour}  OR  {forced labor}  OR  {child labour}  OR  {child labor}  OR  {labour right}  OR  {labor right}  OR  {labour rights}  OR  {labor rights}  OR  {modern slavery}  OR  {human trafficking}  OR  {child soldier}  OR  {child soldiers}  OR  {global jobs}  OR  {living wage}  OR  {minimum wage}  OR  {circular economy}  OR  {inclusive economy}  OR  {rural economy}  OR  {Foreign Development Investment}  OR  {Aid for Trade}  OR  {trade unions}  OR  {trade union}  OR  {working poor}  OR  {Not in Education, Employment, or Training}  OR  {carbon offset}  OR  {carbon offsetting}  OR  {carbon offsets}  OR  {offset project}  OR  {offset projects}  OR  {economic diversification}  OR  {material footprint}  OR  {resource efficiency}  OR  ( {cradle to cradle}  AND  {economy} )  OR  {economic decoupling}  OR  {labour market disparities}  OR  {sustainable tourism}  OR  {ecotourism}  OR  {community-based tourism}  OR  {tourism employment}  OR  {sustainable tourism policy}  OR  {financial access}  OR  {financial inclusion}  OR  {access to banking} )  AND NOT  {health} ) View Less | [399,081](https://08105dxi5-1105-y-https-www-scopus-com.mplbci.ekb.eg/search/history/results.uri?origin=searchhistory&shid=1) |

**WoS**

**- WOS.SCI: 1900 to 2023**

**- WOS.AHCI: 1975 to 2023**

**- WOS.BHCI: 2005 to 2023**

**- WOS.BSCI: 2005 to 2023**

**- WOS.ESCI: 2005 to 2023**

**- WOS.ISTP: 1990 to 2023**

**- WOS.SSCI: 1900 to 2023**

**- WOS.ISSHP: 1990 to 2023**

| # | Search Query | Results |
| --- | --- | --- |
| 1 | (((((((((((((((((((((((((((((((((((((((((((((((((((((((((((((((((TS=(economic growth))  OR TS=(economic development policy))  OR TS=(employment policy))  OR TS=(inclusive economic growth))  OR TS=(sustainable growth))  OR TS=(economic development))  OR TS=(economic globalization))  OR TS=(economic productivity))  OR TS=(low-carbon economy))  OR TS=(inclusive growth))  OR TS=(microfinanc*))  OR TS=(micro-financ*))  OR TS=(micro-credit*))  OR TS=(microcredit*))  OR TS=(equal income))  OR TS=(equal wages))  OR TS=(decent job))  OR TS=(quality job))  OR TS=(job creation))  OR TS=(full employment))  OR TS=(employment protection)) OR TS=(informal employment))  OR TS=(precarious employment))  OR TS=(unemployment))  OR TS=(precarious job))  OR TS=(microenterprise*))  OR TS=(small enterprise))  OR TS=(medium enterprise))  OR TS=(small entrepreneur))  OR TS=(starting entrepreneur))  OR TS=(medium entrepreneur))  OR TS=(social entrepreneurship))  OR TS=(safe working environment))  OR TS=(labor market institution))  OR TS=(forced labour))  OR TS=(child labour))  OR TS=(labour right))  OR TS=(modern slavery))  OR TS=(human trafficking))  OR TS=(child soldier))  OR TS=(global jobs))  OR TS=(living wage))  OR TS=(minimum wage))  OR TS=(circular economy))  OR TS=(inclusive economy))  OR TS=(rural economy))  OR TS=(Foreign Development Investment))  OR TS=(Aid for Trade))  OR TS=(trade unions))  OR TS=(working poor))  OR TS=(carbon offset))  OR TS=(offset project))  OR TS=(economic diversification))  OR TS=(material footprint))  OR TS=(resource efficiency))  OR TS=(economic decoupling))  OR TS=(labour market disparities))  OR TS=(sustainable tourism))  OR TS=(ecotourism))  OR TS=(community-based tourism))  OR TS=(tourism employment))  OR TS=(sustainable tourism policy))  OR TS=(financial access))  OR TS=(financial inclusion))  OR TS=(access to banking)) | 1273162 |
| 2 | ((((((TS=(caries)) OR TS=(dental caries)) OR TS=(dental decay)) OR TS=(dental cavities)) OR TS=(tooth cavities)) OR TS=(tooth deminerali?ation)) OR TS=(enamel deminerali?ation) | 67446 |
| 3 | #2 AND #1 | 669 |

**Pubmed**

| Search number | Query | Results |
| --- | --- | --- |
| 5 | #3 AND #4 | 69 |
| 4 | (((((("Dental Caries"[Mesh]) OR "Tooth Demineralization"[Mesh]) OR (caries[Text Word])) OR (dental decay[Text Word])) OR (dental cavities [Text Word])) OR (tooth cavities[Text Word])) OR (enamel demineralization[Text Word]) | 71,595 |
| 3 | #1 OR #2 | 59,511 |
| 2 | ((((((((((((((((((((((((((((((((((((((((((((((((((((((((economic growth[Text Word]) OR (economic development policy[Text Word])) OR (employment policy[Text Word])) OR (inclusive economic growth[Text Word])) OR (economic globalization[Text Word])) OR (economic productivity[Text Word])) OR (low-carbon economy[Text Word])) OR (inclusive growth[Text Word])) OR (microfinanc*[Text Word])) OR (micro-credit*[Text Word])) OR (equal income[Text Word])) OR (equal wages[Text Word])) OR (decent job[Text Word])) OR (quality job[Text Word])) OR (job creation[Text Word])) OR (full employment[Text Word])) OR (employment protection[Text Word])) OR (informal employment[Text Word])) OR (precarious employment[Text Word])) OR (precarious job[Text Word])) OR (microenterprise*[Text Word])) OR (small enterprise[Text Word])) OR (medium enterprise[Text Word])) OR (small entrepreneur[Text Word])) OR (starting entrepreneur[Text Word])) OR (medium entrepreneur[Text Word])) OR (social entrepreneurship[Text Word])) OR (safe working environment[Text Word])) OR (labor market institution[Text Word])) OR (forced labour[Text Word])) OR (child labour[Text Word])) OR (labour right[Text Word])) OR (modern slavery[Text Word])) OR (child soldier[Text Word])) OR (global jobs[Text Word])) OR (living wage[Text Word])) OR (minimum wage[Text Word])) OR (circular economy[Text Word])) OR (inclusive economy[Text Word])) OR (rural economy[Text Word])) OR (Foreign Development Investment[Text Word])) OR (Aid for Trade[Text Word])) OR (carbon offset[Text Word])) OR (offset project[Text Word])) OR (economic diversification[Text Word])) OR (material footprint[Text Word])) OR (resource efficiency[Text Word])) OR (economic decoupling[Text Word])) OR (labour market disparities[Text Word])) OR (sustainable tourism[Text Word])) OR (ecotourism[Text Word])) OR (community-based tourism[Text Word])) OR (tourism employment[Text Word])) OR (sustainable tourism policy[Text Word])) OR (financial access[Text Word])) OR (financial inclusion[Text Word])) OR (access to banking[Text Word]) | 18,560 |
| 1 | ((((((((("Economic Development"[Mesh]) OR "Sustainable Growth"[Mesh]) OR "Right to Work"[Mesh]) OR "Unemployment"[Mesh]) OR "Small Business"[Mesh]) OR "Human Trafficking"[Mesh]) OR "Labor Unions"[Mesh]) OR "Working Poor"[Mesh]) OR "Resource Allocation"[Mesh]) OR "Banking, Personal"[Mesh] | 43,836 |
